# Effects of transcranial direct current stimulation on modulating executive functions in healthy populations: a systematic review and meta-analysis

**DOI:** 10.3389/fnhum.2024.1485037

**Published:** 2024-12-13

**Authors:** Guopeng You, Xinliang Pan, Jun Li, Shaocong Zhao

**Affiliations:** ^1^Department of Physical Education, Xiamen University of Technology, Xiamen, China; ^2^School of Kinesiology, Beijing Sport University, Beijing, China; ^3^School of Athletic Performance, Shanghai University of Sport, Shanghai, China

**Keywords:** response inhibition, prefrontal cortex, neural activity, stop-signal task, go/nogo task

## Abstract

**Background:**

Conventional research has asserted that cognitive function, particularly, response inhibition, is closely related to the inferior frontal cortex (IFC), dorsolateral prefrontal cortex (DLPFC), or orbital frontal cortex (OFC), which belong to the prefrontal cortex (PFC). Different targets of anodal or cathodal transcranial direct current stimulation (a-tDCS or c-tDCS) would affect the experimental results, but the stimulation of the same brain target would produce inconsistent findings.

**Purpose:**

This study aimed to investigate the effects of a-tDCS and c-tDCS applied over the PFC for healthy populations on reactive and proactive control process compared with sham or no tDCS conditions, as assessed using the Stop-signal task (SST) and Go/NoGo (GNG) task performance.

**Methods:**

This systematic review was performed following the Preferred Reporting Items for Systematic Reviews and Meta-Analysis guidelines. Search was conducted on Web of Science, Google Scholar, PubMed, Elsevier, Scopus, and Science Direct until March 2024. Studies that assessed the inhibitory control in SST or/and GNG tasks were included to achieve a homogenous sample.

**Results:**

Fourteen studies were included for meta-analyses, which were performed for two outcome measures, namely, stop-signal reaction time (SSRT) and commission error (CE) rate. A-tDCS and c-tDCS over the PFC had significant ergogenic effects on SST performance (mean difference = −17.03, 95% CI [−24.62, −9.43], *p* < 0.0001; mean difference = −15.19, 95% CI [−19.82, −10.55], *p* < 0.00001), and that of a-tDCS had a positive effect on GNG task performance (mean difference = −1.42, 95% CI [−2.71, −0.14], *p* = 0.03).

**Conclusion:**

This review confirmed the engagement of PFC tDCS in reactive and proactive inhibitory processes. Future research should increase sample size and implement personalized stimulus protocols.

## 1 Introduction

Response inhibition is an innate capability of human beings to suppress certain already initiated or planned actions as circumstances demand; this cognitive control process was mediated by a distributed neural network. As a brain mechanism in the context of cognitive process, executive functions play a role in optimizing and using cognitive resources to achieve a given goal (Fehring et al., 2019; Mansouri et al., 2009). Given their significance, enhancing executive functions through neuromodulation techniques has gained interest. Transcranial direct current stimulation (tDCS) is a non-invasive and easy-to-use neuromodulation technology; it has polar characteristics and immediate effects, which can induce facilitatory or inhibitory effects on the cerebral cortex by applying low-intensity direct current to a stimulated cortical region (Filmer et al., 2014). A recent study indicated that tDCS is considered a promising adjuvant therapeutic modality for human cognitive functions, such as response inhibition and impulse control (Li et al., 2019a). However, the equivocal and even contradictory cognitive outcome of tDCS in many neuropsychological disorders hampers the progress (Hoy and Fitzgerald, 2010).

Response inhibition was thought to be achieved by reducing the excitability of specific active brain regions and increasing the activity of inhibitory brain regions (Coxon et al., 2006). The ergogenic effects of tDCS on brain-related functioning have received widespread attention in healthy and clinical populations. Intending to effectively improve response inhibition capability, previous studies based on the anode-excitatory cathode-inhibitory physiological model noted that anodal tDCS (a-tDCS) over targeted areas of the brain, such as the dorsolateral prefrontal cortex (DLPFC), supplementary motor area (SMA), and inferior frontal cortex (IFC), can result in a sustained neural drive during manual inhibitory control task (Chen et al., 2021; Gabrielle et al., 2013; Kwon and Kwon, 2013; Sankarasubramanian et al., 2017; Swann et al., 2012; Cunillera et al., 2014). In the present study, cathodal tDCS (c-tDCS) was observed to have an ergogenic effect on cognitive performance (Chen et al., 2021; Schroeder and Plewnia, 2016; Weidacker et al., 2016; Wynn et al., 2019). More than five neuroimaging studies confirmed the neural correlates of response inhibition when using a-tDCS or c-tDCS over stimulation regions (Li et al., 2019b; Sehm et al., 2012; Amadi et al., 2014; Callan et al., 2016; Sankarasubramanian et al., 2017); the results consistently point out changes in functional connectivity and activation beyond the brain networks subserving cognitive control. Nevertheless, the rationale behind utilizing tDCS as a tool in many fields is that subliminal a-tDCS over targeted areas increases the excitability of the cerebral cortex by depolarizing the cell membrane then increasing firing rates, while that of c-tDCS induces the opposite effect by hyperpolarization then decreasing firing rates (Nitsche et al., 2009). Thus, the application of c-tDCS in the context of cognitive control tasks such as Go/NoGo (GNG) task and Stop-signal task (SST), has been questioned due to variable and even contradictory results (Stramaccia et al., 2015; Friehs and Frings, 2019; Dumont et al., 2018). These equivocal issues have appeared in a number of studies utilizing a-tDCS (Perrotta et al., 2021; Friehs and Frings, 2019; Stramaccia et al., 2015). Horvath et al. (2014) proposed that fundamental methodological heterogeneity in the application of tDCS may hinder its internal validity in increasing number of research. The interval time between experimental condition (a-tDCS or/and c-tDCS) and control condition (sham tDCS or no tDCS) is too arbitrary, ranging from a few days to several weeks (Dedoncker et al., 2016). Bell et al. (2020) argued that the differences in individualization and neurochemistry may be related to the variable results. In particular, Li et al. (2006) found gender differences in the effect of tDCS on a cognitive-motor task, and the cerebral cortex of the male showed greater activation compared with the female. Therefore, further optimization of the tDCS testing protocol should consistently reflect the ergogenic effects of tDCS in future studies.

As a probe of response inhibition, GNG and SST in clinical and basic neuroimaging research are increasingly used. GNG is an effective means of evaluating simple reaction-time (RT) tasks, the inhibition of motor response is driven internally (vs. the externally driven in the SST) in this task with a proactive control to a specific target in the inhibitory process (Cunillera et al., 2014; Molero-Chamizo et al., 2018; Weidacker et al., 2016). Participants must respond to frequently presented “Go” signals and withhold the response to “No-go” signals (Zheng et al., 2008), which requires adjusting the response threshold of the former stimulus to balance the go and stop processes. In general, low commission error (CE) rates in “No-go” signals have been interpreted as improved inhibitory control in GNG (Perrotta et al., 2021). In addition, SST is a classic behavioral response inhibition task based on the horse-race model, which is regarded as an elaboration of GNG (Band and Boxtel, 1999). As a typical choice RT task, SST mainly differs from GNG in terms of the time of reactive inhibitory process (Zheng et al., 2008). In SST, participants are required to withhold a response in the “stop process” (e.g., a sound or coloration of the arrow) that has already been triggered in the independent “go process” (e.g., left/right or up/down-pointing arrow) based on a stochastic model. The standard index for reflecting a more efficient inhibitory capacity in SST is the shorter value of the stop-signal reaction time (SSRT) (Logan et al., 1984). All the aforementioned studies report that both tasks can be used to evaluate the effect of tDCS on response inhibition.

This systematic review and meta-analysis has a twofold purpose. The primary aim is to evaluate the effect of a-tDCS and c-tDCS compared with sham-tDCS or no-tDCS on modulating executive functions in healthy populations, respectively. SST and GNG were recruited to sketch the framework by discussing and analyzing cognitive outcome measures considering offline effects. The secondary aim is to determine whether the response inhibition correlates of task performance in SST and GNG can be dissociated.

## 2 Materials and methods

### 2.1 Protocol and registration

This study was performed following the guidelines of the Preferred Reporting Items for Systematic Reviews and Meta-Analysis (PRISMA) for protocols (Moher et al., 2009), and the registration number in PROSPERO is CRD42024556058.

### 2.2 Study selection

The inclusion criteria of this review were as follows: (1) participants must be healthy adults without any psychiatric disorders or musculoskeletal injury; (2) the intervention consisted of at least one session of a-tDCS or/and c-tDCS, and the control group had to be sham-tDCS or no-tDCS; (3) unilateral anodal stimulation, single- or double-blind, randomized experimental design; (4) tDCS overed the prefrontal cortex of the brain; (5) implementation of behavioral tasks, such as SST or/and GNG; (6) measurement of “offline” behavioral changes; (7) English full-text studies published in peer-reviewed journals; and (8) outcome measures should include pre and post stimulation.

Web of Science, Google Scholar, PubMed, Elsevier, Scopus, and Science Direct were searched for appropriate studies published before March 2024. The key words included the following terms: “tDCS” OR “transcranial direct current stimulation” AND “Stop-signal task” OR “Go/no-go task” OR “response inhibition” OR “inhibitory control.” Further relevant studies were manually added by screening the reference sections of retrieved studies, conference papers, and previous reviews.

### 2.3 Data extraction

For each included study, the following data were extracted: (1) study design; (2) sample size and characteristics (gender, age, stimulation interval time); (3) intervention characteristics (duration, current density, electrode size and location); (4) number of sessions; (5) outcome measures (SSRT in the SST, CE rates in GNG); and (6) main results.

### 2.4 Quality assessment

The Physiotherapy Evidence Database (PEDro scale) was utilized to assess study quality because of its proven reliability and validity in evaluating randomized controlled trials and ensure a rigorous analysis of evidence in our systematic review and meta-analysis. Two authors (G.Y. and X.P.) independently rated each included study to assess the quality and risk of bias by using the checklist of the PEDro scale (de Morton, 2009); in case the PEDro scale of a certain study was inconsistent, the third experienced author was consulted to reach a consensus. The original scale contains 11 items, which can be used to determine the external (the first item) and internal (the subsequent criteria) validity of the included studies. The results of the PEDro scale evaluation are shown in Table 1. In the present study, we ignored the first question in the study, because it does not match the present purpose. The PEDro score (0–10) of each item was evaluated as poor (< 4), fair (≥ 4 and ≤ 5), good (≥ 6 and ≤ 8), excellent (≥ 9 and ≤ 10) (de Morton, 2009). The total score of each included study was collected for subsequent statistical analysis.

**Table 1:** Quality assessment of included studies.

Scale	1	2	3	4	5	6	7	8	9	10	11	Total
Bell et al. (2020)	+	+	−	+	+	−	−	+	−	+	+	**7**
Campanella et al. (2017)	+	+	+	+	+	+	−	+	−	+	+	**9**
Campanella et al. (2018)	+	+	+	+	+	+	−	+	−	+	+	**9**
Castro-Meneses et al. (2016)	+	+	+	+	+	−	−	+	+	+	+	**9**
Chen et al. (2021)	+	+	−	+	+	−	+	+	−	+	+	**8**
Cunillera et al. (2014)	+	+	−	+	+	−	−	+	+	+	+	**8**
Dai et al. (2022)	+	+	+	+	+	+	−	+	−	+	+	**9**
Ditye et al. (2012)	+	+	−	+	+	−	−	+	−	+	+	**7**
Fehring et al. (2019)	+	+	+	+	+	−	−	+	+	−	+	**8**
Friehs and Frings (2018)	+	+	−	+	+	−	−	+	+	+	+	**8**
Friehs and Frings (2019)	+	+	−	+	+	−	−	+	−	+	+	**7**
Friehs et al. (2021)	+	+	−	+	+	−	−	+	−	+	+	**7**
Ghayebzadeh et al. (2023)	+	+	−	+	+	−	+	+	−	+	+	**8**
Guo et al. (2022)	+	+	−	+	+	+	−	+	+	+	+	**9**
Guo et al. (2023)	+	+	−	+	+	+	+	+	+	+	+	**9**
Jacobson et al. (2011)	+	+	−	+	+	−	−	+	+	+	+	**8**
Leite et al. (2018)	+	+	−	+	+	−	−	+	+	+	+	**8**
León et al. (2020)	+	+	+	+	+	−	+	+	−	+	+	**9**
Mansouri et al. (2016)	+	+	+	+	+	−	−	+	+	+	+	**9**
Ouellet et al. (2015)	+	+	+	+	+	−	+	+	−	−	+	**9**
Sandrini et al. (2020)	+	+	+	+	+	−	−	+	−	+	+	**8**
Schroeder et al. (2022)	+	+	+	+	+	−	−	+	−	−	+	**7**
Stramaccia et al. (2015)	+	+	+	+	+	−	−	+	−	+	+	**8**
Weidacker et al. (2016)	+	+	−	+	+	−	+	+	+	+	+	**9**

The bold values are obtained by adding the number of “+” in the row.

### 2.5 Statistical analysis

A separate meta-analysis was conducted considering a-tDCS or c-tDCS over the PFC and different tasks for investigating response inhibition by using Review Manager 5.4.1 (Copenhagen: The Nordic Cochrane Centre). Given that some studies used a multi-period stimulation protocol, we decided to extract only the data after the first stimulation. For all included studies requiring quantitative analyses, we entered the three values of mean, SD, and total to calculate the mean difference (MD) and 95% confidence interval (95% CI), which were weighted by inverse variance method using a random-effects model. Study heterogeneity was appraised through the Chi-squared test. The magnitude of the heterogeneity was also quantified using the *I*^2^ statistic, which was estimated as low (< 30%), moderate (≥ 30% and < 50%), substantial (≥ 50% and < 75%), and considerable (≥ 75% and ≤ 100%) heterogeneity (Higgins et al., 2024). In the case of uncertainty in the information and results provided by the authors and when the data could not be obtained from the figures, tables, and results section, a message would be sent to the first author or corresponding author(s) through e-mail or Research gate to request the mean ± SD of the expected result measurement. If the standard error (SE) was reported instead of SD in the studies, we would use a formula ( $SD=SE\sqrt{n}$ , where *n* is the number of participants in each group) to estimate it (Higgins et al., 2024).

## 3 Results

### 3.1 Overall

**Figure 1:**
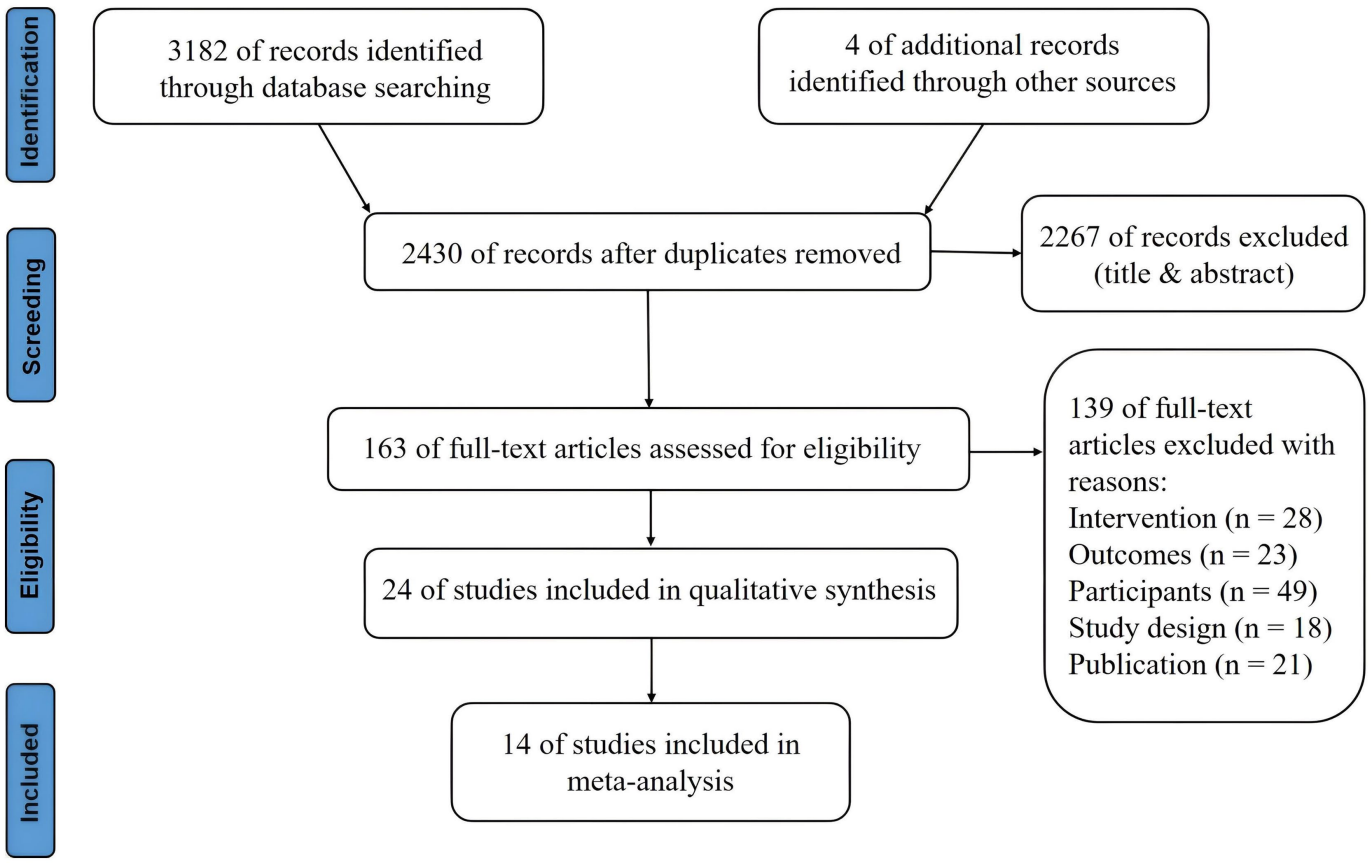
Flow chart of systematic review.

The electronic search yielded a total of 3,182 studies, of which 24 were included in the review. As shown in Figure 1, the selection process is visualized in the PRISMA flow diagram. After the removal of duplicates, 2,430 studies were manually reviewed. A total of 2,267 studies were excluded after assessing titles and abstracts. The full-text articles of the remaining 163 studies were screened based on the eligibility criteria. The 24 included studies comprised a total of 1,228 participants, which were used for qualitative synthesis. Fourteen studies (58.33%) enrolled 714 participants (58.14%) in quantitative synthesis. In the following sections, the effect of a-tDCS and c-tDCS on response inhibition compared with control group would be discussed in each task. The effect of c-tDCS on response inhibition was not used in the meta-analysis because only one eligible study was identified. In addition, the PEDro score of all included studies was between 7 and 9 range, indicating a quality above good level (Table 1).

### 3.2 Study characteristics

Four included studies (16.67%) had a total of 3 (6.67%) (Friehs and Frings, 2019), 5 (10%) (Friehs et al., 2021), 5 (12.5%) (Campanella et al., 2018), and 11 (14.10%) (Schroeder et al., 2022) dropouts. The actual sample sizes per study ranged from 11 to 124 participants (51.17 ± 32.71) aged from 18 to 35 years. The study included 484 males and 620 females, only one study lacked report on the gender of the participants (Bell et al., 2020). Three studies (10.53%) only recruited male participants (Campanella et al., 2017; Campanella et al., 2018; Dai et al., 2022), and one study only recruited female participants (Ghayebzadeh et al., 2023).

As shown in Table 2, a randomized design was performed, 16 (66.67%) were parallel and eight (33.33%) were crossover in all included studies. Twenty-three studies (95.83%) had a sham group as a comparator, and only one study (4.17%) had a no stimulation group as a comparator. The current intensity ranged from 0.5 mA to 2 mA, with a stimulation duration of 20.13 ± 5.82 min (ranging from 10–30 min). In terms of electrode placement, the right PFC was selected as the target area for a-tDCS in all but four studies (Fehring et al., 2019; Mansouri et al., 2016; Dai et al., 2022; Schroeder et al., 2022), which selected the left PFC as the stimulation area. Most c-tDCS was placed over the contralateral supraorbital area.

**Table 2:** Characteristics of the included studies.

Study	Trial design	Task	Number, gender and interval time	Age	Session, duration (min), current density (mA) and electrode size (cm^2^)	Stimulatory electrode and reference	Main results (pre vs. post: ↑or↓; comparison among groups: + or −)
Bell et al. (2020)	Parallel	SST	124, a-tDCS = 62, s-tDCS = 62	N/D	One; 20 min; 1.5 mA; A = 25 cm^2^ and B = 35 cm^2^	Right VLPFC (A) and left supraorbital area (B)	None
Campanella et al. (2018)*	Parallel	GNG	35, a-tDCS = 18, s-tDCS = 17, (M)	20–30	One; 20 min; 2 mA; A = B = 25 cm^2^	Right IFC (A) and left trapezius muscle (B)	A-tDCS ↑
Campanella et al. (2017)*	Parallel	GNG	31, a-tDCS = 15, s-tDCS = 16, (M)	20–30	One; 20 min; 2 mA; A = B = 25 cm^2^	Right IFC (A) and left trapezius muscle (B)	A-tDCS ↑
Castro-Meneses et al. (2016)*	Crossover	SST	A-tDCS = s-tDCS (3 M/11F), 1-7 days	22 ± 3.9	One; 15 min; 1.5 mA; A = B = 25 cm^2^	Right PFC (stimulus) and left cheek (reference)	A-tDCS ↑; a-tDCS (+) vs. s-tDCS
Chen et al. (2021)*	Parallel	SST	92, a-tDCS (15 M/14F), c-tDCS (13 M/17F), s-tDCS (11 M/22F)	17–25	One; 25 min; 1.5 mA; A = B = 35 cm^2^	Right DLPFC (A) and left supraorbital area (B)	A-tDCS ↑, c-tDCS ↑
Cunillera et al. (2014)*	Crossover	SST and GNG	A-tDCS = s-tDCS (4 M/18F), ≥ 7 days	21.2 ± 2.7	One; 18 min; 1.5 mA; A = B = 9 cm^2^	Right IFC (A) and left orbito-frontal cortex (B)	A-tDCS (+) vs. s-tDCS in easy condition
Dai et al. (2022)	Parallel	GNG	29, a-tDCS = 15, c-tDCS = 14, (M)	20–25	one; 30 min; 1.5 mA; A = B = 25 cm^2^	Left DLPFC (A) and right supraorbital area (B)	A-tDCS ↑; c-tDCS ↑
Ditye et al. (2012)	Parallel	SST	22, a-tDCS (3 M/7F), no stimulation (5 M/7F)	23.58 ± 4.16	5 consecutive days; 15 min; 1.5 mA; A = B = 35 cm^2^	Right IFC (A) and left orbitofrontal cortex (B)	None
Fehring et al. (2019)	Crossover	SST	A-tDCS = s-tDCS (36 M/37F), 7 days	18–32	Two; 10 min; 1.5 mA; A = 10 cm^2^ and B = 24 cm^2^	Left DLPFC (A) and right supraorbital area (B)	A-tDCS ↑
Friehs and Frings (2018)*	Crossover	SST	A-tDCS = s-tDCS (21 M/38F)	24.81 ± 3.69	One; 20 min; 0.5 mA; A = 9 cm^2^ and B = 35 cm^2^	Right DLPFC (A) and left deltoid muscle (B)	A-tDCS ↑; a-tDCS (+) vs. s-tDCS
Friehs and Frings (2019)	Parallel	SST	42, c-tDCS (8 M/12F), s-tDCS (3 M/19F)	22.07 ± 2.57	One; 20 min; 0.5 mA; A = 9 cm^2^ and B = 35 cm^2^	Right DLPFC (A) and left deltoid muscle (B)	C-tDCS ↓; c-tDCS (−) vs. s-tDCS
Friehs et al. (2021)*	Parallel	SST	45, anodal DLPFC-cathodal IFC = 15, cathodal DLPFC-anodal IFC = 13, s-tDCS (6 M/39F)	21.47 ± 2.42	One; 20 min; 0.5 mA; A = B = 9 cm^2^	Right DLPFC (A) and right IFC (B)	None
Ghayebzadeh et al. (2023)	Parallel	GNG	24, a-tDCS = 8, c-tDCS = 8, s-tDCS = 8, (F)	28 ± 3.25	One; 20 min; 2 mA; A = B = 35 cm^2^	Right DLPFC (A) and right supraorbital area (B)	A-tDCS ↑; a-tDCS(+) and c-tDCS(+) vs. s-tDCS
Guo et al. (2022)*	Parallel	SST and GNG	92, a-tDCS [right IFG + pre-SMA = 22, right IFG = 24, pre-SMA = 22], s-tDCS = 24, (43 M/49F)	20.58 ± 1.54	One; 20 min; 2.5 mA; HD-tDCS	Right IFG or pre-SMA (A) and Fz, C4, P4, FT10, TP8, FC4 (B)	None
Guo et al. (2023)*	Parallel	SST	94, a-tDCS + training = 24, a-tDCS = 21, s-tDCS + training = 24, s-tDCS = 25,(41 M/53F)	20.88 ± 1.77	10 consecutive days; 20 min; 2.5 mA; HD-tDCS	Right IFG and pre-SMA (A) and Fz, C4, P4, FT10, TP8, FC4 (B)	A-tDCS ↑
Jacobson et al. (2011)*	Crossover	SST	11, a-tDCS = c-tDCS = s-tDCS (3 M/8F), = 7 days	28.3 ± 6.8	One; 10 min; 1 mA; A = B = 25 cm^2^	Right IFC (A) and left orbito-frontal cortex (B)	A-tDCS (+) and c-tDCS (+) vs. s-tDCS
León et al. (2020)	Parallel	SST	61, a-tDCS (15 M/16F), s-tDCS (12 M/18F)	20.75 ± 2.82	One; 20 min; 1.5 mA; A = 9 cm^2^ and B = 35 cm^2^	Right OFC (A) and left trapezium (B)	A-tDCS ↑
Leite et al. (2018)	Crossover	GNG	tDCS = s-tDCS (49 M/11F), ≥ 3 days	21.5 ± 4.5	One; 30 min; 1 mA; A = 35 cm^2^ and B = 100 cm^2^	Right IFC (A) and left IFC (B)	TDCS (+) vs. s-tDCS
Mansouri et al. (2016)	Crossover	SST	A-tDCS = c-tDCS = s-tDCS (12 M/11F), 2–3 min	20–32	One; 10 min; 1 mA; A = 7.5 cm^2^ and B = 20 cm^2^	Left DLPFC (A) and right supraorbital cortex (B)	A-tDCS (+) vs. s-tDCS
Ouellet et al. (2015)*	Parallel	SST	45, left OFC (3 M/12F), right OFC (6 M/9F), s-tDCS (7 M/8F)	25.09 ± 7.10	One; 30 min; 1.5 mA; A = 35 cm^2^ and B = 55.25 cm^2^	Left or right OFC (A) and right or left OFC (B)	None
Sandrini et al. (2020)*	Parallel	SST	30, a-tDCS (7 M/8F), s-tDCS (7 M/8F)	26.5	One; 20 min; 1.5 mA; A = B = 25 cm^2^	Right IFC (A) and left supraorbital area (B)	A-tDCS (+) vs. s-tDCS
Schroeder et al. (2022)	Parallel	SST	67, c-tDCS [left DLPFC (5 M/15F), left IFG (6 M/17F)], s-tDCS (7 M/17F)	22.81 ± 3.45	One; 30 min; 1 mA; HD-tDCS	Left DLPFC or IFG (A) and Fp1, Fz, C3, F7 or AF7, F1, CP1, TP7 (B)	None
Stramaccia et al. (2015)*	Parallel	SST	115, a-tDCS [rIFC (6 M/14F), rDLPFC (3 M/17F)], c-tDCS [rIFC (8 M/12F), rDLPFC (3 M/17F)], s-tDCS (9 M/26F)	23.37 ± 2	One; 20 min; 1.5 mA; A = B = 16 cm^2^	Right IFC or DLPFC (A) and left supraorbital area (B)	A-tDCS (+) vs. s-tDCS over right IFC
Weidacker et al. (2016)	Crossover	GNG	A-tDCS = c-tDCS = s-tDCS (9 M/9F), 2-9 days	22.06 ± 0.98	One; 20 min; 1.5 mA; A = B = 25 cm^2^	Right DLPFC (A) and left biceps (B)	C-tDCS (+) vs. s-tDCS

*included studies for meta-analysis; A, active electrode; R, reference electrode; N/D, not described; OFC, orbitofrontal cortex; DLPFC, dorsolateral prefrontal cortex; IFC, inferior frontal cortex.

### 3.3 Effect of a-tDCS on response inhibition in SST and GNG tasks

**Figure 2:**
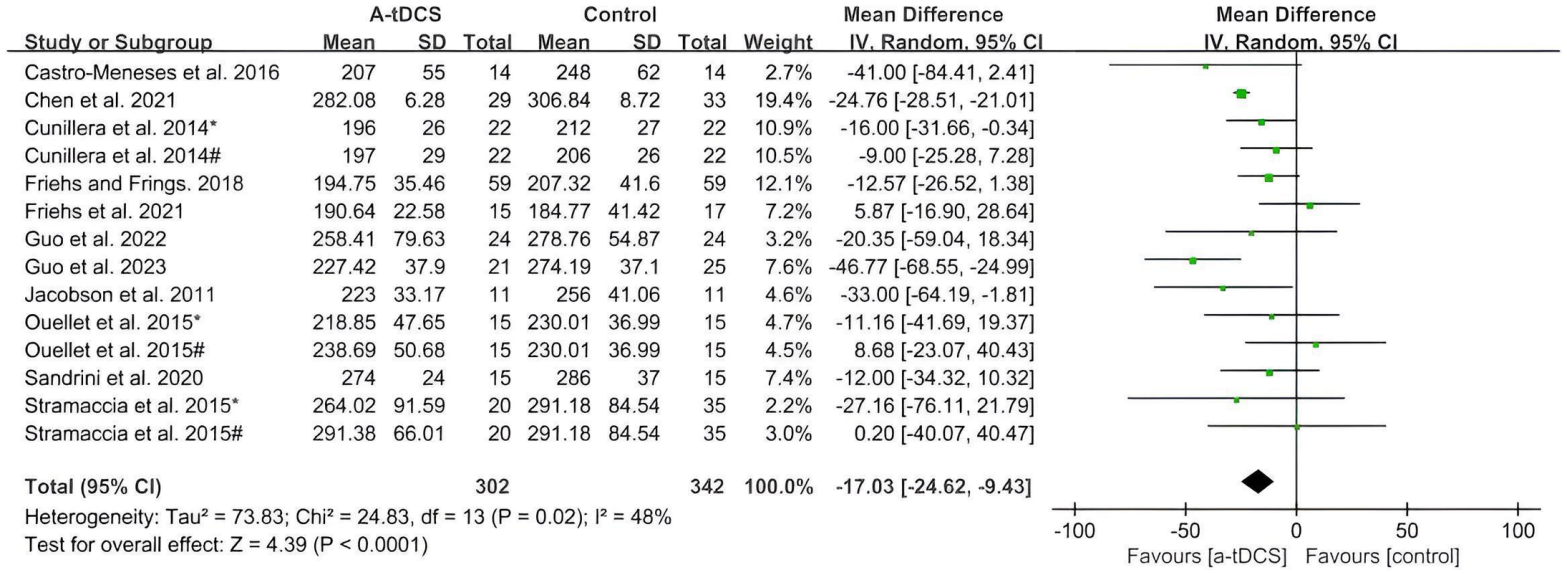
Meta-analysis of a-tDCS on response inhibition in SST task. Cunillera et al. (2014)^*^, easy discrimination condition; Cunillera et al. (2014)^#^, hard discrimination condition; Ouellet et al. (2015)^*^, left OFC group; Ouellet et al. (2015)^#^, right OFC group; Stramaccia et al. (2015)^*^, right IFC group; Stramaccia et al. (2015)^#^, right DLPFC group.

The pooled analysis showed significant changes in response inhibition after single-session a-tDCS compared with the control group in SST and GNG tasks. Figure 2 summarizes the pooled data (size of SSRT) for SST extracted from nine studies on healthy populations. A significant effect in favor of a-tDCS (MD = −17.03 ms; 95%CI = −24.62, −9.43) was found from the random effects model (*p* < 0.0001), with a moderate heterogeneity (Chi^2^ = 24.83, *p* = 0.02 and *I*^2^ = 48%).

**Figure 3:**
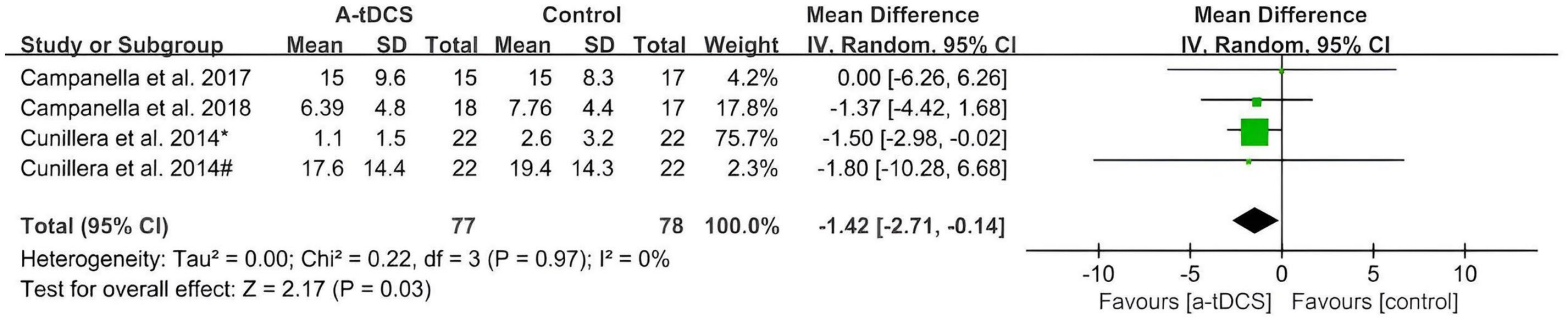
Meta-analysis of a-tDCS on response inhibition in GNG task. Ouellet et al. (2015)^*^, left OFC group; Ouellet et al. (2015)^#^.

As shown in Figure 3, only four studies that analyzed the effect of a-tDCS on response inhibition were found in the GNG task. The pooled MD using the random effects model after a-tDCS was −1.42 (95%CI: −2.71, −0.14, *p* = 0.03), which indicated that the effects were significant with a low heterogeneity (Chi^2^ = 0.22, *p* = 0.97 and *I*^2^ = 0%).

### 3.4 Effect of c-tDCS on response inhibition in SST

**Figure 4:**
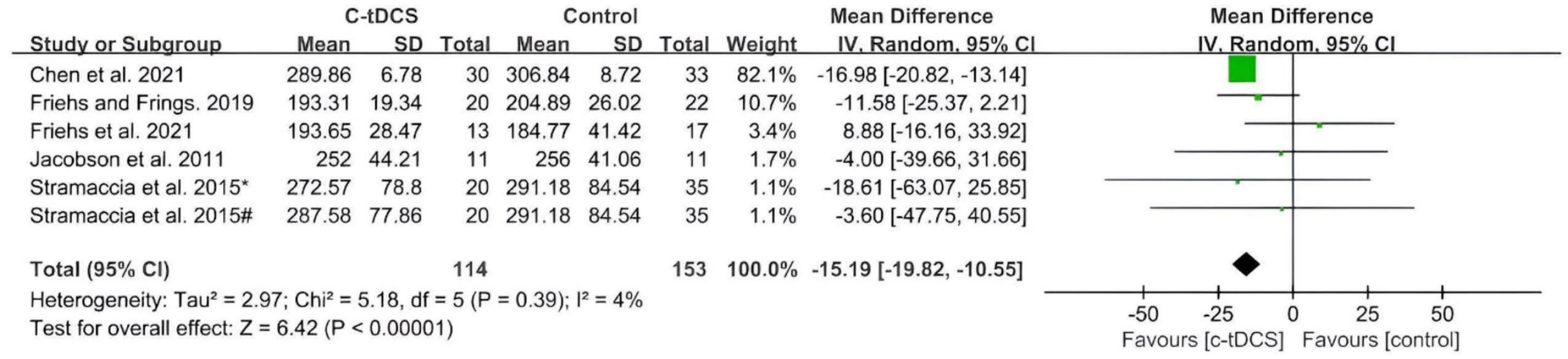
Meta-analysis of c-tDCS on response inhibition in SST task. Stramaccia et al. (2015)^*^, right IFC group; Stramaccia et al. (2015)^#^, right DLPFC group.

The application of single-session c-tDCS over the PFC led to a significant effect at post-intervention compared with control group (Figure 4). The MD using the random effects model was −15.19 (95%CI: −19.82, −10.55, *p* < 0.00001) at post intervention with a low heterogeneity (Chi^2^ = 5.18, *p* = 0.39 and *I*^2^ = 4%).

## 4 Discussion

This systematic review and meta-analysis aimed to determine the efficacy of a-tDCS and c-tDCS over the PFC regions for healthy populations on two common response inhibition tasks. Significant ergogenic effects were detected using tDCS compared with control groups (s-tDCS or no-tDCS) in SST and GNG tasks.

### 4.1 A-tDCS over the PFC

The promising outcomes of a-tDCS on modulation executive functioning tasks have recently attracted attention because of its ergogenic potential. As described previously, the right DLPFC is essential for goal-oriented cognitive control tasks (e.g., SST); related studies reported that a-tDCS over the right DLPFC may effectively improve cognitive inhibition processes (Chen et al., 2021; Friehs and Frings, 2018). A growing amount of evidence-based on fMRI studies has indicated that response inhibition is associated with the continuous activation of the right DLPFC that supports the stop process, especially in SST performance (Hughes et al., 2013; Hughes et al., 2014). Interestingly, a-tDCS over the left DLPFC has been confirmed to have a significant ergogenic effect on performing the cognitive task by comparing within (Fehring et al., 2019) and between groups (Mansouri et al., 2016). These findings proposed that a-tDCS can be used to change the susceptibility of the neurocircuitry to regulate cognitive behavior (Fehring et al., 2019). Additionally, accumulating evidence has shown that the right IFC plays a key role in the SST, and a-tDCS over this region can modulate brain activity and enhance functional connectivity to facilitate response inhibition (Cunillera et al., 2014; Jacobson et al., 2011; Sandrini et al., 2020). Functional neuroimaging studies also demonstrated that the right IFC has a large coincidence with a clear overlapped network for inhibitory processes (Chikazoe et al., 2009). Importantly, a-tDCS over the right IFC combined with cognitive training had a more substantial effect on response inhibition (Ditye et al., 2012). The combination of tDCS and cognitive training may emerge as a new method for enhancing cognitive function. However, in disagreement with the present findings, evidence of a previous meta-analysis showed a null effect on inhibitory control while performing a-tDCS over the right DLPFC (Schroeder et al., 2020). This discrepancy may arise from the fact that the previous study included not only healthy participants but also clinical populations. Additionally, the study found that electrode placement, particularly the position of return electrodes, exhibits distance-dependent effects that influence cortical excitability induction. A study has pointed out that the reverse effect of a-tDCS of PFC was mainly because of the differences in neurochemistry and personality for participants (Bell et al., 2020). On a practical and behavioral level, a study documented that the right IFC a-tDCS had a significantly better effect on response inhibition in a single design compared with the right DLPFC; thus, the right IFC is the most reliable brain stimulation target in SST (Stramaccia et al., 2015). The present meta-analysis demonstrated that in the response domain (e.g., SST), the effects of a-tDCS over the PFC studies were significantly larger than sham/no-tDCS groups with moderate heterogeneity.

As described previously, the effects of unilateral a-tDCS of the right IFC on proactive control were significantly increased compared with sham stimulation by testing the GNG task (Cunillera et al., 2014; Campanella et al., 2017; Leite et al., 2018), which may be due to the fact that the inhibition of the promotion response caused by tDCS was related to the decrease in theta band activity (Jacobson et al., 2012; Campanella et al., 2018). However, the anodal electrode was placed on the right IFC combining with left cathodal IFC tDCS, and its ergogenic effects disappeared. A previous study (Leite et al., 2018) speculated that the left IFC was also a particularly critical region in inhibitory control, and the cathodal electrode would weaken the activity of this region and affect inhibition response. The meta-analysis revealed that the application of a-tDCS over the right IFC for clinical populations (e.g., ADHD, major depression, and obesity) can significantly improve proactive inhibition.

To our knowledge, PFC plays an important role in the cognitive control of behavior. The main contribution of the present study is to show the ergogenic response inhibition effects of PFC a-tDCS on reactive and proactive inhibitory processes by means of the SST and GNG tasks, respectively.

### 4.2 C-tDCS over the PFC

Based on the neurophysiological evidence, cathodal offline tDCS can reduce the glutaminergic neuronal activity and the postsynaptic depolarization is weakened so that the cortex produces a long-term inhibitory effect (Stagg and Nitsche, 2011; Cooke and Bliss, 2006). Compared with a-tDCS in response inhibition, a previous study reported that the effect of c-tDCS was smaller in size (Friehs and Frings, 2018). A study by Friehs and Frings (Friehs and Frings, 2019) further showed that c-tDCS over the right DLPFC can diminish the inhibitory effects. By contrast, another study proved that the a-tDCS and c-tDCS of the right DLPFC had a decrease in SSRT for healthy individuals by testing the SST, which suggested that both were effective for response inhibition (Chen et al., 2021). The former pointed out that the inconsistent results could be due to the difference in study procedures (e.g., electrode size and placement, current density and time) and methodology (pre-post or group-between design) (Friehs and Frings, 2019). The possibility of the latter being able to produce an ergogenic result may be the use of a modified SST. Additionally, the latter believed that the significantly decreased SSRT after c-tDCS was due to its promotion of supraorbital activity and indirect adjustment of related frontal cortex through anatomical connections, such as DLPFC and ACC (Chen et al., 2021). Collectively, studies found that compared with sham stimulation, unilateral c-tDCS over the right IFC can reduce SSRT but did not generate a significant effect in SST (Jacobson et al., 2011; Stramaccia et al., 2015). In the included study, only one determined the positive effect of c-tDCS of the right PFC on inhibition response by testing the GNG task (Weidacker et al., 2016). This study concluded that c-tDCS may contribute to regulating the shift of excitatory glutamate to appropriate levels (Weidacker et al., 2016). According to increased shunting over the scalp, the response inhibition network is mainly contained in the right hemisphere (Menon, 2011; Bari and Robbins, 2013). Combined with the present meta-analysis findings, c-tDCS applied over the right PFC can effectively inhibit executive functions with a low heterogeneity under the premise of increasing the sample size. The findings suggest that c-tDCS over the right PFC may be beneficial for clinical populations with inhibitory control deficits. By reducing glutamatergic activity, c-tDCS could enhance response inhibition and could be a potential therapeutic approach for conditions characterized by impaired cognitive control.

### 4.3 Limitations

Several limitations of this study should be noted. First of all, the participants included in the studies were all healthy populations. Second, the meta-analysis did not perform subgroup analysis in specific regions of PFC. Third, this study did not make a detailed comparative discussion and analysis of stimulation parameters (e.g., current density, duration) and potential moderators (e.g., age, gender). Future studies should investigate the optimal interval time between experimental and control conditions to determine its effect on modulation effectiveness. Finally, the extracted output data were all unilateral offline tDCS after a single session. Future works should use bilateral online intervention to explore the long-term effects of tDCS.

## 5 Conclusion

This systematic review and meta-analysis can reveal the effects of tDCS on modulating executive functions in healthy populations. A-tDCS and c-tDCS can have significant ergogenic effects on SST and GNG tasks, with low to moderate heterogeneity. Specifically, this study affirmed the potentially reactive inhibitory effect of a/c-tDCS in the PFC by evaluating SSRT in SST, and the proactive inhibitory effect of a-tDCS by evaluating CE in GNG.

## Data Availability

The original contributions presented in the study are included in the article/supplementary material, further inquiries can be directed to the corresponding author.
